# Phenomenological Characteristics of Autobiographical Memories: Responsiveness to an Induced Negative Mood State in Those With and Without a Previous History of Depression

**DOI:** 10.5709/acp-0190-8

**Published:** 2016-06-30

**Authors:** Andrew E. P. Mitchell

**Affiliations:** Faculty of Health and Social Care, University of Chester, UK

**Keywords:** autobiographical memory, history of depression, negative mood state, phenomenological characteristics

## Abstract

In this study we investigated the relative accessibility of phenomenological characteristics in autobiographical memories of 104 students with and without a previous history of a depression. Participants recalled personal events that were elicited with cue words and then asked to rate these personal events for a number of phenomenological characteristics. The characteristics were typicality, rumination, valence, importance of others, expectancy, desirability, and personal importance. The effects of previous history of depression (without history or with previous history of depression) and self-reported mood (pre- and post-negative mood induction) on autobiographical recall was examined by employing a mixed factor design. Self-reported mood was measured as a manipulation check, before and after Mood Induction Procedure. Typicality, rumination and personal importance showed significant interaction effects in those with a history of depression. Ordinal regression supported the finding that those with a history of depression had a higher chance of typicality and personal importance than those without a history of depression. The results indicate that recall of autobiographical characteristics is in part dependent on induced negative mood state and on previous history of depression. The findings may prompt future research into targeted interventions that reduce individual tendencies for heightened cognitive reactivity in negative mood states for those with a history of depression.

## Introduction

 The aim of this present study is to investigate the effects of negative mood on the phenomenology of autobiographical memories in those participants with a history of depression versus those with no history of depression. It was predicted that those with a history of depression would show autobiographical memory reactivity in a negative mood state. This was predicted due to the effects of cognitive reactivity ( Lau, Haigh, Christensen, Segal, & Taube-Schiff, 2012) and ongoing vulnerability due to a history of depression (O’Grady, Tennen, & Armeli, 2010). The term ‘phenomenology of autobiographical memory’ refers to the subjective experience of memories (e.g., whether the memory is rated as vivid, detailed, and emotionally intense), and is assessed by participants using a Likert response. Cognitive models of emotional disorders suggest that depressed individuals show bias in memory and a tendency to interpret ambiguous information in a negative way (Peckham, McHugh, & Otto, 2010). To date, there is a limited understanding of the interplay between memory, interpretation of ambiguous information, and previous history of depression. It has been suggested that memory and interpretation are possibly affected in those with a current depression, showing a greater increase in negative cognitive bias than those who are not depressed (Werner-Seidler & Moulds, 2011). It is unclear, however, whether phenomenological characteristics in those who have a previous history of depression but no signs of current depression, show mood effects in an induced negative mood state. 

 The term autobiographical memory refers to an individual’s record of experiences from their personal life in the form of an internal life story (Williams et al., 2007). Autobiographical memory is hierarchically organised (Conway & Pleydell-Pearce, 2000) in an interlinked network of increasing sensory detail, vividness and perceptual qualities, and affective information, mood and emotional aspects, associated with the memory event. Brewer (1996) made a distinction between phenomenally experienced, recollective memory, and factually known autobiographical memories. The phenomenological characteristics that are most frequently investigated in literature are vividness, sensory details, and valence. The two most widely used scales, amongst a few that exist, are the Autobiographical Memories Questionnaire (AMQ; Rubin, Schrauf, & Greenberg, 2003) and the Memory Experiences Questionnaire (MEQ; Sutin & Robins, 2007; Luchetti & Sutin, 2015) which share most but not all the dominant characteristics. 

 There have been a number of theories put forward to explain the differences seen in autobiographical memory. One such explanation is cognitive reactivity, the ease with which characteristic patterns of thinking are reactivated by mild changes in negative mood is related to relapse into depressive states (Lau et al., 2012). It would appear that during past depressive episodes an association is formed between depressed mood and dysphoric content such that future depressed mood will reactivate corresponding patterns of thinking ( Joiner & Rudd, 2000). Mood state effects on autobiographical recall can be nicely explained from the *affect-as-information model* (Schwarz, 2001). The theory suggests that people use transient feeling states as information for making evaluation calls about specific events. For instance, when recalling an autobiographical event, individuals ask themselves during a general search “What was my life like at that time?” This answer is informed by the present mood state and Schwarz’s (2001) research indicates that people who are in a negative mood state will make more negatively biased assessments. 

 Memory bias, when accompanied by negative mood state, seems to affect memory recall in the direction of negatively biased events, which has been suggested to be a stable phenomenon in a depressive episode and possibly continues in remission from depression (Williams et al., 1996 , for a review). Scher, Ingham, and Segal (2005) suggest that bias in interpretation and memory are correlates of depression, but the bias in memory might only be detected in a depressive state and not during remission from depression. Scher et al. (2005) suggested that bias in memory might not be accessible in those without current episode of depression, due to the lack of a current negative mood state. The present research is interested in the effects of negative mood state on the phenomenological characteristics of autobiographical memories in individuals with and without a history of depression. 

 As the above theoretical models of autobiographical memory, cognitive reactivity and affect-as-information model do not explicitly address the phenomenological characteristics, a literature search pertaining to these characteristics was undertaken. Previous researchers suggested that negative mood affects cognitive content such as valence and emotionality of memories (Scher et al., 2005 , for a review). Researchers have explicitly looked at specific phenomenological characteristics of memory; for example, memories rated high in typicality are events in memory that are like other events that are recalled (Heaps & Nash, 2001). Heaps and Nash (2001) identified that true event memories were less typical. Evidence suggests that due to the overgeneral memory that is seen in depression, typicality might increase because of the frequent reiterations that could be due to the hierarchical nature of autobiographical memory (Conway, 1996). Such a hierarchy would suggest that general memories are accessed more frequently than specific events. Williams et al. (2007) suggested that because of the frequency of access to general memories compared to specific memories, the latter are less easily recalled. 

 The AMQ (Rubin, Schrauf, & Greenberg, 2003) contains an item for rehearsal of an event. In depression, rumination, which is the repeated rehearsal of negative content (van Vreeswijk & de Wilde, 2004), has been identified as a central feature in depression. The present study included the item “How often do you think about this event?” to identify rumination/rehearsal. Williams, Barnhofer, Crane, and Duggun (2006) suggested that ruminative style is encouraged by negative mood, which takes up working memory resources and results in overgeneral memories. The MEQ (Sutin & Robins, 2007) has an item on valence or so called emotional tone. The retrieval of emotionally congruent memories has been noted in negative mood states (Walker, Skowronski, Gibbons, Vogl, & Thompson, 2003). 

 Away from the two main scales, which do not attempt to cover the full range of phenomenological experiences in the literature, two items were included from Ross and Wilson (2002) who asked participants to evaluate the personal importance of the memory event and importance of others (i.e., acquaintances). The most personally important experiences seem to result in the strongest memories (Clore & Schnall, 2005). Also, as humans experience negative mood, the importance of others might seem to matter more, as do the details of the world around them (Holland & Kensinger, 2010). Again, away from the big measures of phenomenological characteristics, an item was selected on expectancy due to findings that depression impairs a person’s ability to imagine specific scenarios in the future (Williams et al., 1996). Mood seems to affect record of past experience, which in turn affects expectancy and ability to imagine the future (Williams et al., 1996). Furthermore, Singer (1990) investigated the relationship between autobiographical memories and desirability of the event. Negative mood lowers individual engagement with environment and ratings of social context, which in turn affects desirability (Ryan & Deci, 2000). 

 The aim of the present study is to evoke autobiographical memories using cue words and then to ask participants to rate each memory event on a number of characteristics using an analogue scale. Single-items from the AMQ and MEQ, and items used in previous studies (Heaps & Nash, 2001; Ross & Wilson, 2002; Singer, 1990) were selected to assess typicality, rumination, valence, importance of others, expectancy, desirability, and personal importance. It is known that participants with a history of depression rate their memories as less accessible in negative mood (Werner-Seidler & Moulds, 2011). It was hypothesized that there would be a difference in those with and without a history of depression following a mood induction. An interaction between history and mood was predicted with an increase in scores, for typicality, rumination, importance of others, expectancy and personal importance. For valence and desirability, it was hypothesized that those with a history of depression would show an interaction between history and mood, with a decrease in scores. The present study constructed single item questions to assess the phenomenological characteristics from established questionnaires assessing the phenomena (see Table 1). The advantage of using a single-item measure, rather than the preferred multiple-item measure, in research involving mood are down to the short-lived nature of induced mood states. Single items have the advantage that they are less time consuming as only a certain number of questions can be posed before the temporary mood state dissipates. It is acknowledged that other phenomenological characteristics assessed in the literature, such as visual perspective and/or distancing, may have mood state effects, but they were not considered in the present study. Nonetheless, the questions asked are theoretically motivated and cover a breadth of contemporary research in phenomenological autobiographical memory. 

**Table 1.** Single Item Dimensions and Mapping for Phenomenological Characteristics

Phenomenological characteristic	Item	Literature for chosen phenomenological item
Typicality	How typical is the event in your life?	Heaps & Nash (2001)
Rumination / rehearsal	How often do you think about it?	Rubin, Schrauf, & Greenberg (2003)
Valence	How did you feel about the event at the time?	Sutin & Robins (2007)
Importance of other people	How important were other people in the event?	Ross & Wilson (2002)
Expectancy	Was the event expected?	Williams, Ellis, Tyers, Healy, Rose, & MacLeod (1996)
Desirability	How desirable was the event?	Singer (1990)
Importance	How important was the event?	Heaps & Nash (2001)

In the present study, the effects of an induced negative mood on phenomenological characteristics were observed during a cued autobiographical memory task. Participants self-reporting a history of depression were expected to show effects of induced negative mood on autobiographical characteristics. Participants rated a number of phenomenological characteristics, and the endorsements of these characteristics were observed pre- and post-mood induction. It was hypothesized that there would be an interaction between history and mood in those with a history of depression for the phenomenological characteristics. If a negative mood manipulation in those with a previous history of depression alters phenomenological characteristics, then a focus on coping strategies that directly target mood state effects on autobiographical recall might benefit this group of individuals.

## Method

### Participants

 A total of 104 University students (12% male and 88% female) participated in the present study (*M*_age_ = 28.5 years, *SD* = 3.54, range 26-31). Twenty-seven participants self-reported a previous history and 77 participants reported no previous history for depression. They were tested in groups of 20 to 34 people. A post hoc power analysis was conducted using the software package, GPower (Faul, Erdfelder, Lang, & Buchner, 2007). The sample size of 104 was used for the statistical power analyses. Cohen’s (1977) recommended effect sizes were as follows: small (*f* = .1), medium (*f* = .25), and large (*f* = .4). The alpha level used for this analysis was *p* < .05. The post hoc analyses revealed the statistical power for this study was .52 for detecting a small effect, .99 for detecting a moderate effect, >.99 for detecting a large effect size. Thus, there was more than adequate power (i.e., .80) at the moderate to large effect size level, but less than adequate statistical power at the small effect size level. 

### Materials

#### Beck Depression Inventory

 The Beck Depression Inventory (BDI-II; Beck, Steer, & Brown, 1996) was used to measure participants’ level of depressive symptoms on a 0-3 Likert scale, with higher numbers corresponding to more severe symptoms. The 21- item, self-report questionnaire assesses symptoms present within the past two weeks. Scores range from 0 to 63. It was utilized to assess the level of depressive symptoms. Participants scoring BDI-II > 12 were excluded from the study for their protection. Twelve participants were excluded for this reason. The BDI-II > 12 cut-off is recommended for non-clinical undergraduate students by Dozois, Dobson, and Ahnberg (1998) . The BDI-II demonstrated good reliability with a Cronbach’s α = .72. The participants were non-clinical undergraduate students who self-reported a history of depression, thus, any other comorbid mental health problems cannot be excluded, but the BDI-II was utilized to screen out current depression and perhaps any other comorbid conditions within this group. 

#### History of previous depression

 The history of depression questionnaire was used as a proxy to assess previous history of depression. Participants were asked to self-report whether they had previously been prescribed treatment for symptoms of depression. Those who had received previous treatments, such as pharmacology, cognitive behavioural therapy or counselling, were considered to have a history of depression and those who had not and were without a current history were classified as without a history of depression. The present investigation has selected participants according to a self-reported history of depression rather than a current diagnosis of depression. The self-reported history of depression has limitations due to the accuracy of self-reports but has the advantage in that it limits the time involved to have a clinician led diagnostic assessment and has been successfully used to identify depressive history (McChargue & Cook, 2007). Additionally, the researcher was interested in assessing depressive history over a much longer time interval, rather than a shorter “snapshot” for a current diagnosis of depression. The examination of depression over a longer timeframe allows for the “aggregation” of depressive history and is more likely to reveal mood state effects due the reinforcement of those very effects during these previous episodes. 

#### Mood State Assessment

 The University of Wales Institute of Science and Technology Mood Adjective Checklist - UMACL (Matthews, Jones, & Chamberlain, 1990) was selected due to its ability to assess general non-clinical mood states. The UMACL measure consists of a 29-adjective checklist containing three factorial scales of hedonic tone (HT), tense arousal (TA), and energetic arousal (EA). Scores on the hedonic tone and energetic arousal scale are negatively related to the level of negative mood (low-scaled scores represent greater negative mood), whereas higher values reflect a greater negative mood on the tense arousal scale. Hedonic tone items included happy, dissatisfied, cheerful, sorry, depressed, satisfied, sad, and contented. Tense arousal items included relaxed, nervous, tense, jittery, composed, restful, and calm. Energetic arousal items included items energetic, alert, passive, sluggish, vigorous, unenterprising, active, and tiredness on a 4 point Likert scale. The UMACL was chosen due to its sensitivity to external stressors (Matthews et al., 1990). The psychometric properties were identified for the UMACL and shown to have good internal reliability for non-clinical mood variations (Matthews et al., 1990). Cronbach’s α for the subscales were 0.78, 0.83, and 0.89, respectively. 

#### Cued Autobiographical Memory

 The autobiographical memory cuing technique as used by Williams and Broadbent (1986) was modified to assess autobiographical content on presentation of words as memory cues. The modified version of the Autobiographical Memory Test (AMT; Williams & Broadbent, 1986) has been widely used to assess autobiographical content. Eight neutral cue words were drawn from words used in previous research (Dalgleish, Cameron, Power, & Bond, 1995; Janssen, Chessa, & Murre, 2005), with similar properties for concreteness and frequency. Four neutral cue words were used pre-mood induction and four different neutral cue words were used post-mood induction. The experimenter read each word as it was displayed on the projector after two trial runs at obtaining a specific memory. Participants were instructed to report the first specific personal memory triggered by each stimulus word and write down as much detail as necessary, and they were prompted to bring it to a close after approximately 1 min. Participants were instructed to rate each of the memories on a 7-point Likert scale ranging from 1 (*not at all*) to 7 (*very much*), with 4 as a *neutral rating*. Items included typicality, rumination, valence, importance of others, expectancy, desirability, and personal importance of the event (see Table 1). 

#### Automatic Thought Questionnaire

 The ATQ-30 (Hollon & Kendall, 1980) was administered to act as a self-focused manipulation for frequency of negative cognitions. Participants rated 30 questions on a 5-point Likert scale indicating frequency (1 = *not at all*, 5 = *all the time*). The frequency of negative cognitions was not part of the analysis due to the main focus on the interaction between history and mood state. 

#### Mood Induction

 The intervention phase utilized the Negative Mood Induction Procedure (NMIP; Velten, 1968). This procedure involves participants reading a list of 60 graduated depressogenic self-referent statements. Example items included statement one, “Today is neither better nor worse than any other day”, to statement 60 which states, “I want to go to sleep and never wake up”. The current research project administered the Velten technique to small groups of approximately 20-34 participants. The participants were asked to read each statement shown for approximately 10 s while projected on a white screen. The meta-analytical effect of *r* = .52 for negative mood induction was established (Westermann, Spies, Stahl, & Hesse, 1996). 

### Design and Procedure

The study was approved by the institution’s ethical committee. The participant information sheet indicated a negative mood induction technique was involved and that participation could be withdrawn at any time. All participants signed a consent form prior to completing the assessment scales. Those participants that were depressed were actively screened out and appropriate sign posting for support for depression was provided. Positive mood induction was undertaken after the procedure, along with a full debriefing.

 After providing informed consent, participants were asked to complete the UMACL (Matthews et al., 1990). The mood measure provided the baseline mood scores for the study and was used as a mood predictor variable. The following was provided on each information sheet: “You will be given a list of words which describe the moods or feelings which people have. To complete the checklist, you should indicate how well the word describes how you feel at the moment (and not just how you usually feel). You must choose one of four possible replies—‘definitely’, ‘slightly’, ‘slightly not’, and ‘definitely not’.” These choices are numbered from 1 (*definitely*) to 4 (*definitely not*), respectively. Following the UMACL, participants completed the ATQ-30 and Cued Autobiographical Memory procedure. Participants were instructed to report the first specific personal memory triggered by each stimulus word and write down as much detail as necessary. Following the four cue words, the participants were given a scoring sheet to rate each phenomenological characteristic. 

Between pre-mood induction and post-mood induction, the negative mood induction procedure was used to induce a negative mood state. Participants read each NMIP sentence one by one with the following instruction: “Remain silent, and try to experience each statement as though it is happening to you.” Post-mood induction, the same participant group was re-administered the UMACL, ATQ-30, and the cued autobiographical memory task. The entire procedure lasted about an hour.

### Data Analysis

Analyses of variance (ANOVAs) were utilized to investigate the difference between the pre-test and post-test means as a function of previous history of depression. From pre- to post-induction, mood ratings on UMACL changed in accordance with the induced negative mood. The mean and SD for all scores was calculated and a manipulation check for mood was conducted, as this was a pre-condition for testing the mood effects on autobiographical memory characteristics. Check for outliers and incorrect data input was undertaken. There were no outlier or extreme scores observed in each outcome variable. The data was normally distributed. The paired sample *t*-test was used to test the difference between pre- and post-mood induction scores for each sub-scale on the UMACL. Significance level of *p* < .05 was adopted. SPSS (IBM Corp v21, 2012) Bonferroni sequential adjusted p-values are quoted. Cohen’s *d* have been reported with all statistically significant values and effect sizes organised into values for Cohen’s *d* effect sizes. Accordingly, *d* < 0.20 represents a negligible effect, *d* > 0.2 to < 0.5 a small effect and *d* > 0.8 a large effect.

To determine whether the changes in the recalled autobiographical memories were influenced by history of depression, a 2 (time; pre-mood rating, post-mood rating; within-subject factor) × 2 (group; with a previous history of depression, without a previous history of depression; between-subjects factor) mixed ANOVA was conducted to observe any interaction effects.

### Results and Discussion

#### Manipulation check

The UMACL was used for subjective evaluation in this study, both as a general indicator of mood state and, more specifically, to evaluate changes in energetic arousal, tense arousal, and hedonic tone. The outcomes of statistical analyses are reported in Table 2. At the start of the experiment, there were no significant group differences in mood between those with a history and those without a history of depression. Lowered energetic arousal equals less active and alert. Increased tense arousal is consistent with being more anxious and nervous. Lowered hedonic tone equals loss of interest and diminished pleasure response. It was expected that negative mood induction would result in a reduction in energetic arousal and hedonic tone, and conversely an increase in tense arousal.

There was a significant difference between pre-manipulation and post-manipulation mood ratings on the UMACL. The results indicated that energetic arousal and hedonic tone showed significant increases in negative mood (i.e., reductions in mean scores as expected), *t*(99) = 6.09, *p* < .001, Cohen’s *d* = 0.61, and *t*(89) = 5.66, *p* < .001, *d* = 0.60, respectively. There was no increase in tense arousal, *t*(95) = -0.99, *p* = .33, *d* = - 0.10. The explanation for the result observed with tense arousal may be due to the test-retest effect—that is, tense arousal measures anxiety, which may increase less than expected due to the retest being a more familiar task and counteracting the effects of the increase in anxiety due to the mood induction procedure.

**Table 2.** Independent *T*-Test for Equality of Means Pre- and Post-Mood Induction

	Pre-Mood Induction			Post-Mood Induction	
	Without	With		Without	With	
	(*n* = 77)	(*n* = 27)		(*n* = 77)	(*n* = 27)	
	*M* (*SD*)	*M* (*SD*)	*t*-test	*M* (*SD*)	*M* (*SD*)	*t*-test
EA	22.7 (3.6)	22.1 (3.3)	0.66 *ns*	20.3 (4.1)	18.7 (4.8)	1.65 *ns*
TA	14.0 (3.8)	15.5 (4.3)	- 1.66 *ns*	14.3 (4.2)	17.3 (4.3)	- 2.88 *
HT	27.8 (3.4)	26.4 (3.6)	1.75 *ns*	24.9 (4.8)	23.3 (4.6)	1.43 *ns*

*Note*. * *p* < .005, *M*, mean; *SD*, standard deviation; EA, energetic arousal; TA, tense arousal; HT, hedonic tone; Without, without a history of depression. With, with a history of depression.

#### Mixed Repeated-Measures ANOVA

It was hypothesized that there would be a difference in those with (*n* = 27) and without (*n* = 77) a history of depression for the phenomenological characteristics. To test the specific hypotheses a mixed effects ANOVA was completed, predicting an interaction with a larger increase in typicality, rumination, importance of others, expectancy, personal importance, and a decrease in valence and desirability in those with a history of depression but not in those without a previous history.

A significant interaction was observed between time and group in their effects on typicality, *F*(1, 99) = 4.68, *p* = .03, *d* = 0.44, such that the previous history of depression group experienced a larger increase in typicality from pre- to post-manipulation compared to the group without history of depression. Planned contrasts were conducted in order to determine the nature of the interaction. Planned contrasts indicated that the previous history of depression group experienced a significant increase, *t*(25) = -5.30, *p* < .001, *d* = -1.06, while the without history group also experienced a significant change from pre- to post-manipulation, *t*(75) = -4.73, *p* < .001, *d* = -0.55. The observed interaction between time and group indicates that both groups experienced an increase in typicality post-mood induction. A significant main effect was observed for time on typicality endorsements, *F*(1, 99) = 48.49, *p* < .001, *d* = 1.41, but the main effect for previous history of depression on typicality endorsements was non-significant, *F*(1, 99) = 0.30, *p* = .59, *d* = 0.11.

A significant interaction was observed between time and group in their effects on rumination, *F*(1, 99) = 49.78, *p* < .001, *d* = 1.43, such that the group with a previous history of depression experienced a larger increase in rumination from pre- to post-manipulation compared to the group without history of depression. Planned contrasts indicated that the group with previous history of depression experienced a significant increase, *t*(25) = -6.34, *p* < .001, *d* = -1.27, while the without history group also experienced a significant change from pre- to post-manipulation, *t*(75) = -5.89, *p* < .001, *d* = -0.68. A non-significant main effect was observed for time on rumination, *F*(1, 99) = 0.33, *p* = .57, *d* = 0.12, and the main effect for the previous history of depression group on rumination was also non-significant, *F*(1, 99) = 0.35, *p* = .56, *d* = 0.12.

A significant interaction was observed between time and group in their effects on personal importance, *F*(1, 99) = 4.80, *p* = .03, *d* = 0.44, such that the previous history of depression group experienced a larger increase in personal importance from pre- to post-manipulation compared to the without history of depression group. Planned contrasts indicated that with previous the group with history of depression experienced a significant increase, *t*(25) = -2.71, *p* = .01, *d* = -0.54, while the group without history experienced no significant change from pre- to post-manipulation, *t*(75) = 0.29, *p* = .78, *d* = 0.03. A non-significant main effect was observed for time on personal importance endorsements, *F*(1, 99) = 3.57, *p* = .06, *d* = 0.38, and the main effect for the previous history of depression group on personal importance was also non-significant, *F*(1, 99) = 0.29, *p* = .59, *d* = 0.11. There were no other main or interaction effects observed for valence, importance of other people, expectancy, and desirability.

#### Regression model for self-reported history of depression

To determine whether phenomenological characteristics were associated with induced negative mood, ordinal regressions were undertaken, post- on pre-manipulation, for each phenomenological characteristic. The model for self-reported history of depression shows a goodness of fit for typicality and personal importance with pseudo r2 of 3.8% and 5.5% respectively, indicating the amount of information gained when including the predictors in the model in comparison to the intercept-only model was greater. For pre- to post-mood induction for typicality, the model suggests that those with a history of depression had a higher chance of typicality than those with no history of depression, *OR*: 0.45; 95% CI [-1.578, -.006], *p* = .048. For pre- to post-mood induction in personal importance, the model suggests that those with a history of depression had a higher chance of personal importance than those without a history of depression, *OR*: 0.39; 95% CI [-1.744, -.169, *p* = .018. Rumination, valence, importance of others, expectancy, and desirability characteristics failed to show statistical significance in the model. The results of the regression analyses are reported in Table 4.

**Table 3.** Independent *T*-Test for Equality of Means Pre- and Post-Mood Induction

	Pre-Mood Induction			Post-Mood Induction		
	Without	With		Without	With	
	(*n* = 77)	(*n* = 27)		(*n* = 77)	(*n* = 27)	
	*M* (*SD*)	*M* (*SD*)	*t*-test	*M* (*SD*)	*M* (*SD*)	*t*-test
Typicality #	3.1 (1.5)	2.6 (1.1)	1.65 *ns*	4.2 (1.4)	4.5 (1.4)	-0.97 *ns*
Rumination #	3.5 (1.4)	3.2 (1.0)	0.86 *ns*	4.8 (1.2)	4.8 (1.5)	0.04 *ns*
Valence	5.1 (1.2)	5.0 (1.1)	0.42 *ns*	4.8 (1.5)	4.9 (1.5)	-0.55 *ns*
Other people	5.3 (1.4)	5.1 (1.2)	0.80 *ns*	5.5 (1.5)	5.6 (1.0)	-0.32 *ns*
Expected	4.3 (1.6)	4.4 (1.3)	-0.18 *ns*	4.4 (1.5)	4.2 (1.7)	0.54 *ns*
Desirable	4.5 (1.4)	4.8 (1.3)	-1.10 *ns*	4.6 (1.7)	4.8 (1.6)	-0.69 *ns*
Important #	5.0 (1.4)	4.7 (1.3)	0.91 *ns*	5.0 (1.6)	5.6 (1.0)	-2.03 *

Note. * *p* < .05; #, interaction effect between history and mood; *M*, mean; *SD*, standard deviation; Without, without a history of depression; With; with a history of depression

**Table 4.** Ordinal Regression of Post- on Pre-Characteristic for Group With Self-Reported History of Depression

	Typicality	Rumination	Valence	Importance of others	Expected	Desirable	Importance
Non-Vulnerable							
Cumulative logit	-4.45	-4.57	-4.48	-4.59	-4.65	-4.68	-4.43
Cumulative odds [exp(Cum.logit)]	0.01	0.01	0.01	0.01	0.01	0.01	0.01
Vulnerable							
Cumulative logit	-5.24	-4.70	-5.06	-4.66	-4.48	-4.43	-5.38
Cumulative odds [exp(Cum.logit)]	0.01	0.01	0.01	0.01	0.01	0.01	0.01
Odds Ratio (With/Without)	0.45	0.88	0.56	0.93	1.19	1.28	0.39
Odds Ratio (Without/With)	2.21	1.14	1.79	1.07	0.84	0.78	2.59
Model Fit	< 0.05*	0.73	0.16	0.87	0.66	0.55	< 0.02*
Goodness-of-Fit	0.10	0.22	0.04	1.22	0.33	0.22	0.06
Pseudo R2 ‘Nagelkerke’	0.04	< 0.001	0.02	< 0.001	< 0.001	< 0.001	0.06
Coefficient	-0.79	-0.13	-0.58	-0.07	0.18	0.24	-0.95
*SE*	0.40	0.40	0.40	0.40	0.40	0.40	0.40
*p*-value	< 0.05*	0.74	0.15	0.86	0.66	0.54	< 0.02*
95% CI	-1.578 to -0.006	-.903 to .644	-1.362 to .201	-.845 to .708	-.599 to .949	-1.531 to 1.019	-1.744 to -.160

*Note*. CI, confidence interval; SE, standard error; Without, without a history of depression; With, with a history of depression

 It is possible that pre-existing differences, beyond mood induction, underlie some of the observed results. Table 3 suggests that there may have been some pre-existing differences that did not reach the level of significance in the individual characteristics. Significant interaction effects in the individual characteristics may reflect such a-priori differences, at least partly independent from mood induction, consistent with effects reported in the work of Scherrer and Dobson (2009) . However, the significant interaction on typicality, rumination, and personal importance is consistent with previous studies, suggesting mood induced changes (Holland & Kensinger, 2010). This is further supported by the ordinal regression for typicality and personal importance, which might be more sensitive to detect different effects of mood on self-reported history of depressive symptoms, but ordinal regression was non-significant for rumination. 

## General Discussion

 The study described here was designed to explore the effects of mood manipulation on the phenomenological characteristics of cued autobiographical memories. The effects of a previous history of depression (without history or with history of depression) and self-reported mood (pre- or post-negative mood induction) on autobiographical recall was measured. The hypothesis that there would be an interaction between history of depression and mood following a negative mood induction received some support. The key significant phenomenological characteristics that showed an interaction with a previous history of depression were typicality, rumination, and personal importance. This was supported by the ordinal regression showing that those with a history of depression had a higher chance of typicality and personal importance than those without a history of depression. The findings indicate that people with a history of depression do have mood effects on some of the phenomenological characteristics measured in this study, as compared to those without a previous history of depression, suggesting that induced negative mood state combined with previous history of depression may be responsible. The finding is consistent with recent research showing a pattern of cognitive reactivity that is temporarily dependent on levels of sadness in those vulnerable to depression, whereas individuals less vulnerable to depression are not (Clasen, Fisher, & Beevers, 2015). Those individuals with a history of depression have shown mood-state dependent cognitive effects, including preferential memory for mood-congruent information and unhelpful depressogenic attitudes (Robinson, Watkins, & Harmon-Jones, 2013 , for a review). 

The mood induction succeeded and was effective in eliciting negative mood with lower energetic arousal and hedonic tone. These components suggested a lack of energy, sluggishness and tiredness states (low energetic arousal); and loss of interest and diminished pleasure response (low hedonic tone). The third mood component was in the expected direction but was non-significant. As the main hedonic tone (valence dimension) and energetic arousal (arousal dimension) had both shown significant changes, we concluded that an overall increase in negative mood had occurred, according to the expected mood manipulation.

The present study specifically investigated the difference between pre- and post-mood induction as a function of previous history of depression. The evidence from the present study suggests that those with a history do show changes in cognitive characteristics for typicality, rumination, and personal importance under transient low mood, while those without a history show increased negative mood without the change in phenomenological characteristics. Those with a history of depression may have particular cognitive characteristics that are triggered by negative mood state which influence their cognitive processes. This formulation closely matches the cognitive therapy model in which negative mood has the potential to activate latent cognitions in vulnerable individuals which have been primed in previous depressive episodes.

 The phenomenological characteristics that showed no significant interaction effects were valence, importance of others, expectancy, and desirability. These four phenomenological content characteristics contrast with the phenomenological process characteristics such as rumination in autobiographical memory. Further research that focusses on content and process characteristics in autobiographical memory seems warranted. However, the non-signiﬁcant inﬂuences on valence, importance of others, expectancy, and desirability characteristics are perplexing. These findings were not in line with the hypothesis that predicted a significant interaction effect in all chosen phenomenological characteristics in those with a previous history of depression. They are also in contrast to previous cognitive reactivity research which generally found an association between negative mood and cognitive reactivity (Timbremont & Braet, 2004). These authors found increased reactivity for valence in those with current depression. The study criteria utilized a diagnosis of depression rather than a history, which may account for the discrepant findings. These inconsistencies warrant some consideration. Another possible explanation is that the present study induced a negative mood state rather than investigating those with a depressed mood state. It is noted that most of the literature regarding autobiographical memory has focused on those currently experiencing depressed symptoms (Gotlib & Neubauer, 2000) as in Timbremont and Braet’s (2004) study. This might account for the unexpected observation and suggests that more research is needed to disentangle effects of depression, history of depression, and induced temporary negative mood states. 

 From a theoretical perspective, the current study provides evidence partially supporting that it is a transient mood state combined with a previous history of depression that is important in producing a change in phenomenological characteristics. It might be that the temporary induced negative mood state triggers and activates content that matches the mood state or has previously been associated with the mood state, in those with a history of depression. Another theory is that the protective elements of non-congruent memories become disengaged, thereby allowing the full effects of negative mood to be felt by the individual. The findings provide some support for the *affect-as-information model* (Schwarz, 2001), with results indicating that transient mood states effect evaluation calls about events from the past. This must remain tentative as this was not tested directly but only concluded from the changes of phenomenological characteristics pre- to post-mood induction. 

### Limitations

The main challenge of research is balancing the ability to maintain ecological validity while maintaining control over memory retrieval. The limitations of the present design include the acknowledgement that the components of the procedure, such as filling out questionnaires and repeated testing, may have affected the findings. There is a possibility that the findings reflected experimenter-elicited demand effects. It is acknowledged that the design is sensitive to internal validity problems due to maturation and history effects which would have been better controlled by a control group or a neutral induction.

 Furthermore, the present research focused on sad mood induction, rather than positive mood induction. The focus of the research was in part influenced by the differential activation hypothesis (Teasdale, 1983), which suggests that previous depressed individuals as opposed to never depressed individuals can be distinguished by the degree of activation of sad cognitions during sad mood. However, future research would gain valuable insights into positive mood induction with phenomenological changes, given that research has highlighted differences in positive memories (Werner-Seidler & Moulds, 2011). 

The present study was designed to examine induced negative mood effects in those with a history of depression that might be analogous to those that occur in previous cases of unipolar depression, but the researcher cannot exclude instances of depression that might include periods of euphoria. The present study investigated history of depressive mood rather than any specific diagnosis, such as bipolar disorder, thus, caution is warranted in generalizing to any specific diagnostic group.

Future research would need to remedy these limitations and specifically recruit individuals with a history of depression based on diagnostic criteria, with equal numbers of participants with and without previous diagnosis of depression. Future research could build in checks to determine if participants follow instructions as provided. Notwithstanding these limitations and necessary caution in interpretations, the effects of mood induction were robust, as validated in a manipulation check with a careful measurement of mood before applying the mood induction and then measuring it again after the induction.

### Conclusions

 The findings, regarded with appropriate caution and aware of a need for replication, might lead to future research into effective intervention strategies for those with a history of depression. This may be achieved through targeted approaches that reduce individual tendencies for heightened cognitive reactivity in negative mood states (Segal et al., 2006). Although, this is not a new approach, it could help clients to observe and monitor cognitive content when they feel sad and highlight that extra diligence that may be required when in sad mood. Clinicians could teach patients to monitor their mood and learn strategies that address ‘outside in’ approaches seen in behavioural activation strategies (Manos, Kanter, & Busch, 2010). Metacognition techniques could also be taught to help patients disengage from the unhelpful phenomenological content reactivated by mild changes in negative mood state (Ma & Teasdale, 2004). 

In summary, the overall findings from this study suggest that some differences exist between those with and those without a history of depression which were only observable in induced negative mood state. The evidence from this study supports theory that, in addition to previous history of depression, a current negative mood state is a necessary condition to measure and observe a change in phenomenological content. It would seem that cognitive reactivity is not merely related to previous history of depression but is partly dependent on negative mood, if a history of depression is given.
